# A comparative analysis of bacterial community characterization and host–bacteria interactions between bi-macroalgal blooms caused by *Ulva prolifera* and *Sargassum horneri*

**DOI:** 10.3389/fmicb.2025.1728378

**Published:** 2025-12-18

**Authors:** Yu Zang, Xiaoxue Liu, Song Xue, Lei Yin, Shiliang Fan, Xiaoxiang Miao, Mingzhu Fu, Jie Xiao, Zongling Wang

**Affiliations:** 1 Research Center of Marine Ecology, First Institute of Oceanography, Ministry of Natural Resources, Qingdao, China; 2 College of Animal Science and Technology, Yangzhou University, Yangzhou, China

**Keywords:** bacterial assembly, green tide, macroalgae–bacteria interactions, metabolites, microbial diversity, *Sargassum horneri*, *Ulva prolifera*

## Abstract

Macroalgal blooms have increasingly occurred in coastal regions worldwide. Since 2017, simultaneous green tides (*Ulva prolifera*) and golden tides (*Sargassum horneri*) have recurred annually in the Yellow Sea, forming a unique large-scale bi-macroalgal bloom. Interactions between macroalgae and their associated bacterial communities are recognized as key ecological drivers of algal bloom dynamics. In this study, the differences in phycosphere-associated bacterial communities and algae-derived metabolites between *U. prolifera* and *S. horneri* were explored using the 16S rRNA amplicon combined with broad-spectrum metabolomics. The results reveal that the diversity of phycospheric and epiphytic bacterial communities of *S. horneri* is significantly higher than that of *U. prolifera*. We observed distinct phycosphere bacterial recruitment between the two macroalgal species. Verrucomicrobiae were the stable core microbiota in the *U. prolifera* phycosphere, whereas Gammaproteobacteria and Bacteroidia represented the core members in that of *S. horneri*. Community assembly analyses indicate that deterministic processes predominantly shape the epiphytic bacterial communities, suggesting strong host selection effects. Metabolomic profiling further revealed that the metabolites secreted by *U. prolifera*, such as phenolic acids and organic acids, promote the proliferation and colonization of *Verrucomicrobiae Rubritalea*, which may enhance the stress tolerance of the host. In contrast, the amino acids, nucleotides, lipids, and their derivatives are key metabolites that promote the colonization of Gammaproteobacteria *Vibrio* and *Marinomonas* on the *S. horneri* surface, which may inhibit host growth through the production of algicidal substances. Together, these results suggest that *U. prolifera* and *S. horneri* can secrete different metabolites that recruit epiphytic microbial communities and influence macroalgae–bacteria interactions. These findings provide insights into the ecological mechanisms underlying host–bacteria interactions and their roles in the formation and persistence of macroalgal blooms.

## 1 Introduction

Large-scale macroalgal blooms, such as green tides and golden tides, have been increasingly observed in coastal regions around the world (Smetacek and Zingone, 2013). These blooms are often driven by eutrophication and other anthropogenic activities, and they have profound ecological impacts by altering community structures and affecting primary productivity (Lyons et al., 2014; Li H. et al., 2025). Since 2007, green tides caused by *Ulva prolifera* have been occurring continuously in the Yellow Sea; these tides represent the world’s largest blooms due to their massive floating biomass and large geographical distribution (Xiong et al., 2023). Notably, large-scale floating *Sargassum horneri*, known as a golden tide, was observed in the same region during the green tide outbreak in 2017 (Xiao et al., 2020). Since then, green and golden tides have recurred together annually from spring to summer, forming a unique massive bi-macroalgal bloom. Field surveys have indicated that, during the early development of green tides (i.e., from late April to May), *U. prolifera* accumulated in the southern Yellow Sea and subsequently drifted northward along the coast. Meanwhile, *S. horneri* exhibited a significant spring bloom in the East China Sea and subsequently extended into the southern Yellow Sea between March and May, where it eventually senesced and underwent natural sinking. These observations indicate that the occurrence of these two types of macroalgal blooms has unique characteristics in time and space. The development and demise of such blooms are influenced by a combination of environmental and biological factors (Seyedsayamdost et al., 2011). The interactions between microorganisms (i.e., “biological factors”) and algal bloom species, as well as their potential regulatory roles in the progression of algal blooms (Seymour et al., 2017; Yue et al., 2025), are gradually being evaluated. Accordingly, it is crucial to elucidate the interactions between macroalgae and associated microbial communities to better understand the underlying mechanisms that regulate the dynamics of macroalgal blooms.

Macroalgae–bacteria interactions represent a fundamental ecological relationship in aquatic environments. These interactions primarily occur in microscale, either on the thallus surface or within the area surrounding the thallus (i.e., the phycosphere), which are multifarious with interactions ranging from mutualistic to competitive (Greff et al., 2017; Kessler et al., 2018). Mutualistic relationships between macroalgae and bacteria are often based on the exchange of resources (Liu et al., 2023). For example, the fucoidan, agarose, and cellulose-degrading activities of Gammaproteobacteria, Bacteroidetes, and Actinobacteria allow them to direct the acquisition of their carbon demand and energy from brown algae (Bakunina et al., 2000; Hehemann et al., 2012; Sichert et al., 2020). In turn, numerous symbiotic bacteria promote host macroalgal growth by producing essential macronutrients or facilitating nutrient supply (Helliwell et al., 2011; Ghaderiardakani et al., 2017). Diverse bacteria within the phycosphere, such as those found in *Laminaria digitata*, can contribute to the host’s nutrient exchange and protection from pathogenic microorganisms (Foster et al., 2011; Jackson et al., 2022). Furthermore, macroalgae–bacteria interactions can enhance the algae’s access to key micronutrients, such as iron and vitamins (Croft et al., 2005). However, bacteria can also have inhibitory effects on algae via competitive or antagonistic relationships, for example, through competition for nutrients or the production of algicidal compounds (Mayali and Azam, 2004; Seymour et al., 2017). For example, Proteobacteria *pseudoalteromonas* infects diatoms *Skeletonema costatum* and causes cell lysis via extracellular proteases (Mitsutani et al., 2001). Some benthic algae efficiently utilize available nitrogen, thereby negatively impacting ammonia-oxidizing bacteria (Risgaard-Petersen et al., 2004). Therefore, understanding macroalgae–bacteria interactions is crucial for elucidating macroalgae community dynamics, which might be closely associated with the development and persistence of macroalgal blooms.

Although not yet systematically studied, there is evidence to support the hypothesis that macroalgae–bacteria interactions influence the dynamics of macroalgal blooms. Specifically, the community composition of the algae-associated bacteria dynamically changes with bloom development (Zhou et al., 2020a), and phycosphere bacteria exhibit clear host specificity among different macroalgal taxa (Aires et al., 2016). The host-specificity of bacterial communities might be due to extracellular secondary metabolites and physicochemical properties of algal secretions, which can induce bacteria responsible for metabolizing these compounds (Matsuo et al., 2005). The composition of algal exudates is closely related to the physiological state of the thallus. During growth phases, the thallus releases soluble, low-molecular-weight molecules, such as amino acids, carbohydrates, and organic acids (Bjørrisen, 1988). A majority of these metabolites have strong chemoattractant effects that facilitate bacterial colonization (Seymour et al., 2010). When thalli senesce or decay, high-molecular-weight molecules, such as polysaccharides, proteins, nucleic acids, and lipids, are released through exudation or cell lysis, thereby providing carbon-rich substrates that create a unique biochemical microenvironment (Seymour et al., 2010). Therefore, such algae-derived metabolites have potential implications for the phycosphere-residing bacteria and colonizers. Despite increasing recognition of the close association between algae-bacteria interactions and bloom dynamics, their specific roles in the development of green and golden tides remain poorly understood. Furthermore, the assembly of bacterial communities during algal blooms involves complex ecological mechanisms influenced by deterministic and stochastic processes. Phycospheric conditions and biological interactions can modulate these processes, ultimately affecting microbial diversity and succession patterns (Dini-Andreote et al., 2015). Experiments with many diatom blooms have demonstrated that algal surface community assembly processes are mediated by both the bloom-forming species and colonizing bacteria (Mönnich et al., 2020). However, it is unclear whether these traits can be generalized to other disaster-causing species, particularly those that may be in macroalgal blooms.

In this study, we investigated the variations in phycosphere-associated bacterial communities and extracellular metabolites between *U. prolifera* and *S. horneri* during the bi-macroalgal blooms in the southern Yellow Sea. We integrated 16S rRNA amplicon sequencing with broad-spectrum metabolomics to characterize and compare phycospheric communities and to identify algal-secreted metabolites. The main objectives of the study were as follows: (1) to compare the composition and diversity of epiphytic and phycosphere-associated bacterial communities between *U. prolifera* and *S. horneri*; (2) to reveal the factors affecting the assembly processes of phycosphere-associated bacterial communities in *U. prolifera* and *S. horneri* blooms; and (3) to explore the role of vital metabolites secreted by host macroalgae in regulating the variations in phycosphere bacterial communities. Finally, further exploration of macroalgae–bacteria interactions across various macroalgal blooms will provide new insights into the ecological functions of these interactions and the regulatory mechanisms underlying macroalgal bloom dynamics.

## 2 Materials and methods

### 2.1 Sample collection and treatment

**Figure 1:**
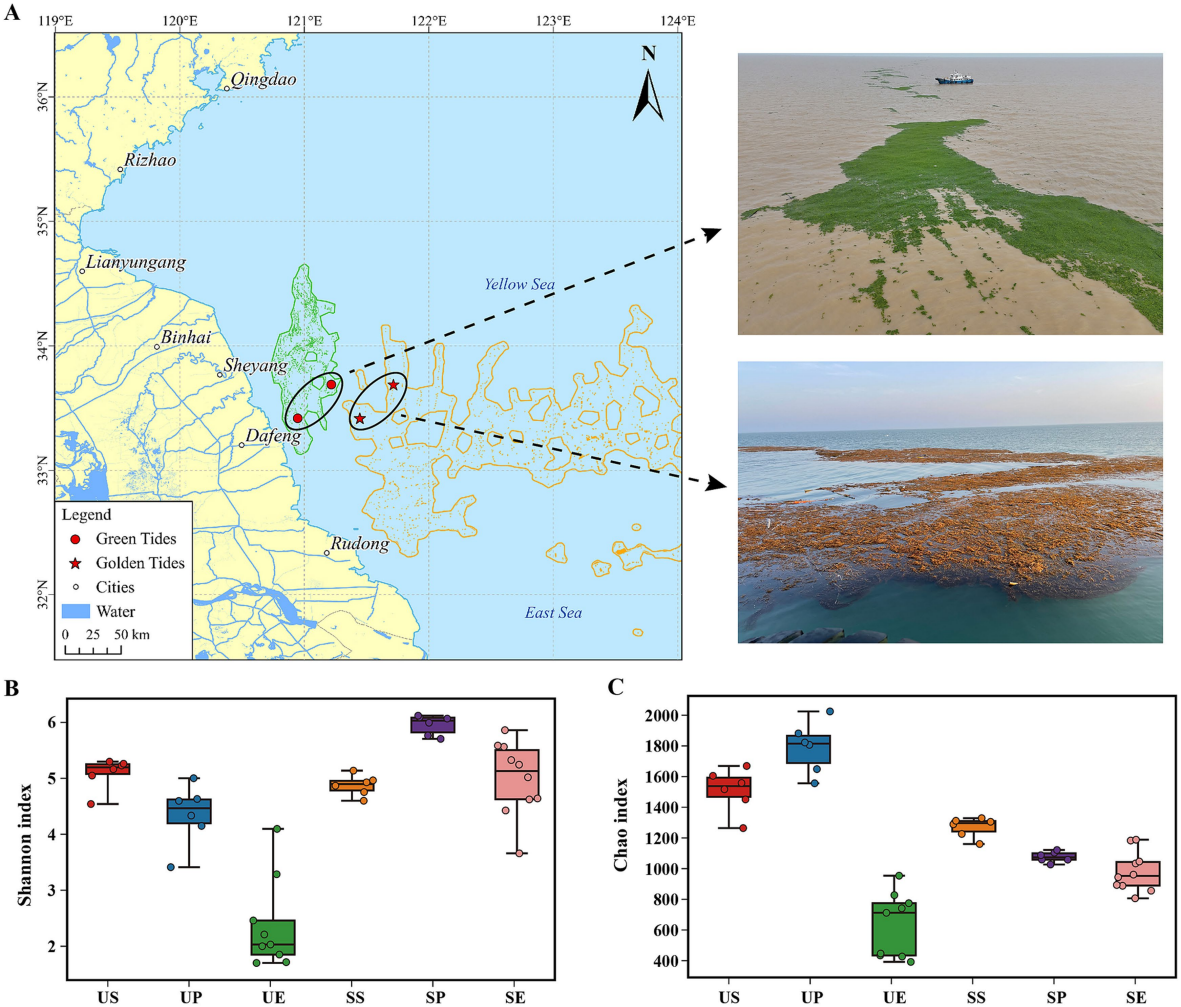
Distribution of sampling sites and comparisons of bacterial community diversity between the *U. prolifera* and *S. horneri*. **(A)** Geographic distribution of the sampling sites and field conditions during both algal blooms. The distribution data were obtained from the interpretation and analysis of remote sensing satellite HY-1E data, and the imaging time was on 13 May 2024. **(B)** Shannon and **(C)** Chao indices of bacterial communities. US: *U. prolifera* surrounding seawater; UP: *U. prolifera* phycosphere; UE: *U. prolifera* epiphytic bacteria; SS: *S. horneri* surrounding seawater; SP: *S. horneri* phycosphere; SE: *S. horneri* epiphytic bacteria.

A field tracking investigation was conducted based on real-time remote sensing and field observations of *U. prolifera* and *S. horneri* blooms on 13 May 2024. Two sampling stations were selected in each macroalgal bloom area for sample collection (Figure 1A). The samples were collected at different stages: *U. prolifera* in the pre-bloom stage and *S. horneri* in the decline stage. Seawater samples (from both blooms’ outbreak areas and non-algae-covered areas) and algal thalli samples were collected, with 3–5 biological replicates for each sample type and region. The bacterial samples of the phycosphere and surrounding seawater were collected from a depth range of 0–20 cm using a sterilized hydrophore, as described by Liu X. et al. (2024). A total of 1.5 L of seawater was sequentially filtered through 3-μm and 0.22-μm polycarbonate filters to concentrate the bacterial cells. The epiphytic bacteria were obtained from *U. prolifera* and *S. horneri* samples according to an established method (Qu et al., 2021). The algal samples were rinsed in autoclaved artificial seawater and then vortexed with sterilized silica sand. All vortex suspensions produced after three rounds were combined and prefiltered through a 0.8-μm membrane to remove debris, and the bacteria were collected on a 0.22-μm polycarbonate filter. All filters were stored at −80 °C until DNA extraction.

### 2.2 Measurement of environmental properties

*In situ* temperature, pH, salinity, and dissolved oxygen (DO) of the phycosphere and surrounding seawater were measured using a YSI ProQuatro handheld multiparameter meter (Xylem, Inc., Yellow Springs, OH, USA). Seawater samples were filtered through 0.45-μm membranes. The filtrates were collected using polyethylene bottles at −20 °C for further laboratory nutrient analysis. Nutrient concentrations, such as phosphate (PO_4_^3−^), nitrate (NO_3_^−^), nitrite (NO_2_^−^), ammonium (NH_4_^+^), and silicate (SiO_3_^2−^), were measured using a QuAAtro automatic analyzer (Seal Analytical Ltd., King’s Lynn, UK).

### 2.3 DNA extraction and sequence analysis

Microbial DNA was extracted according to the manufacturer’s instructions, and the 16S rDNA target region of the ribosomal RNA gene was amplified by PCR using primers 341F (5′-CCTACGGGNGGCWGCAG-3′) and 806R (5′GGACTACHVGGGTWTCTAAT3′) (Guo et al., 2017). At the end, 2 × 250 bp paired-end reads were generated by sequencing on the Novaseq 6000 platform, and raw reads were further filtered using FASTP (v. 0.18.0). Using the FLASH program (v. 1.2.11), paired reads were overlapped to generate raw tags, which were then filtered under specific conditions to obtain high-quality clean tags (Bokulich et al., 2013). Chloroplast and mitochondria sequences were excluded from the datasets. A total of 5,562,691 raw reads and 5,523,952 clean sequence reads were obtained. The clean tags were subsequently clustered into operational taxonomic units (OTUs) at ≥97% similarity using UPARSE (v. 9.2.64). The representative OTU sequences were classified into organisms using a naive Bayesian model with an Ribosomal Database Project (RDP) classifier (v. 2.2) based on the SILVA database (v. 138.1).

### 2.4 Determination of metabolome of algae surface exudates and analysis

Surface exudates from *U. prolifera* and *S. horneri* were collected using a modified version of the procedure described by Hu et al. (2018). Briefly, algal surfaces were gently wiped with sterile cotton swabs and rinsed 5–6 times with sterile seawater to separate impurities and epiphytic bacteria. For each replicate, 50 g samples were submerged in 250 mL of sterile phosphate-buffered saline (PBS, 10×) and then incubated for 8 h at a temperature consistent with the sampling site environment. Then, the exudate solution was collected, filtered through a 0.22-μm membrane, and stored in centrifuge tubes at −80 °C for subsequent metabolomic analysis.

The metabolomic profiling of algae surface exudates was performed using an ultra-performance liquid chromatography-electrospray ionization-tandem mass spectrometry system (UPLC-ESI-MS/MS) system (UPLC, SHIMADZU Nexera X2, Kyoto, Japan). The analytical conditions used in this study followed the protocol described by Liu H. et al. (2024). Metabolites were separated using an Agilent SB-C18 column (2.1 × 100 mm, 1.8 μm) with a 9-min linear gradient. The effluent was subsequently introduced into an ESI triple quadrupole-linear ion trap mass spectrometer (QTRAP-MS) for precise qualitative and relative quantitative analyses. Metabolites were identified by matching characteristic multiple reaction monitoring (MRM) data with the corresponding retention times, and relative quantification was based on MRM peak area comparisons among samples. Metabolite annotation and classification were performed using the self-built MetWare database (MWDB; MetWare Co., Wuhan, China). Principal component analysis (PCA) was conducted in R^1^ using the “prcomp” function to visualize differences in exudate metabolite profiles between *U. prolifera* and *S. horneri*. The variable importance in projection (VIP) was used to assess each metabolite’s contribution to group differentiation. The differential metabolites were screened based on the criteria of VIP ≥ 1, |Log_2_FC (fold change)| ≥ 1, and subsequently enriched in the KEGG pathway to elucidate functional differences in metabolic profiles between the two species. Pathway enrichment was considered statistically significant when the *p-*value was < 0.05.

### 2.5 Statistical analysis

The geographical distribution of the sampling sites was constructed using ArcGIS Pro (v. 3.5.3). Alpha diversity was assessed by computing the Shannon and Chao indices via Mothur (v. 1.30.2). Principal coordinates analysis (PCoA) of the Bray–Curtis distance was generated using the “vegan” package and plotted using the “ggplot2” package (v. 3.3.5). A stacked bar plot of the community composition was also visualized using the “ggplot2” package. Biomarker features in each group were screened using LEfSe software (v. 1.0), and a ternary plot of species abundance was prepared using the “ggtern” package. The Mantel test was used to detect correlations between the bacterial community structure and environmental variables. βNTI was employed in combination with the Bray–Curtis-based Raup-Crick metric (RCbray) to quantify the contribution of major ecological processes to the assembly of bacterial communities (Stegen et al., 2013). The percentages were derived from the statistical averages of the ecological process results. Averaged bacteria community-level niche width represented by Levins’ niche width index was determined using the “spaa” package in R (v.4.3.1). To further infer the life history strategies of the bacterial communities associated with both algae, we estimated the OTU abundance-weighted average 16S rRNA gene copy number for each sample. The rRNA operon copy number for each OTU was estimated from the rrnDB database based on the known rRNA operon copy numbers of close relatives. The specific calculations were performed according to the previous method (Wang et al., 2025). All data in this study are presented as mean ± standard deviation (SD) for three replicates. Statistical analysis was conducted using IBM SPSS 25.0 software. The differences between groups were identified using one-way analysis of variance (ANOVA) with the significance level set as a *p*-value of < 0.05. The least significant difference (LSD) test was used for pairwise comparisons.

## 3 Results

### 3.1 Diversity differences of bacterial communities between *Ulva prolifera* and *Sargassum horneri*

Our results revealed substantial differences in the diversity of bacterial communities between *U. prolifera* and *S. horneri*. The *U. prolifera* phycosphere exhibited a higher Chao index but lower Shannon index compared to its surrounding seawater, whereas the *S. horneri* phycosphere showed a higher Shannon but lower Chao index relative to its surrounding seawater (*p* < 0.01) (Figures 1B,C). Moreover, the *α* diversity of both macroalgae’s epiphytic bacteria was lower than their respective phycospheric communities, and *U. prolifera* epiphytes were significantly lower than *S. horneri* epiphytes (*p* < 0.01). PCoA based on Bray–Curtis distances revealed that the distributions of epiphytic and phycospheric bacteria between *U. prolifera* and *S. horneri* were relatively dispersed (*p* < 0.01) (Supplementary Figure S1A).

### 3.2 Bacterial community structure differences between *Ulva prolifera* and *Sargassum horneri*

**Figure 2:**
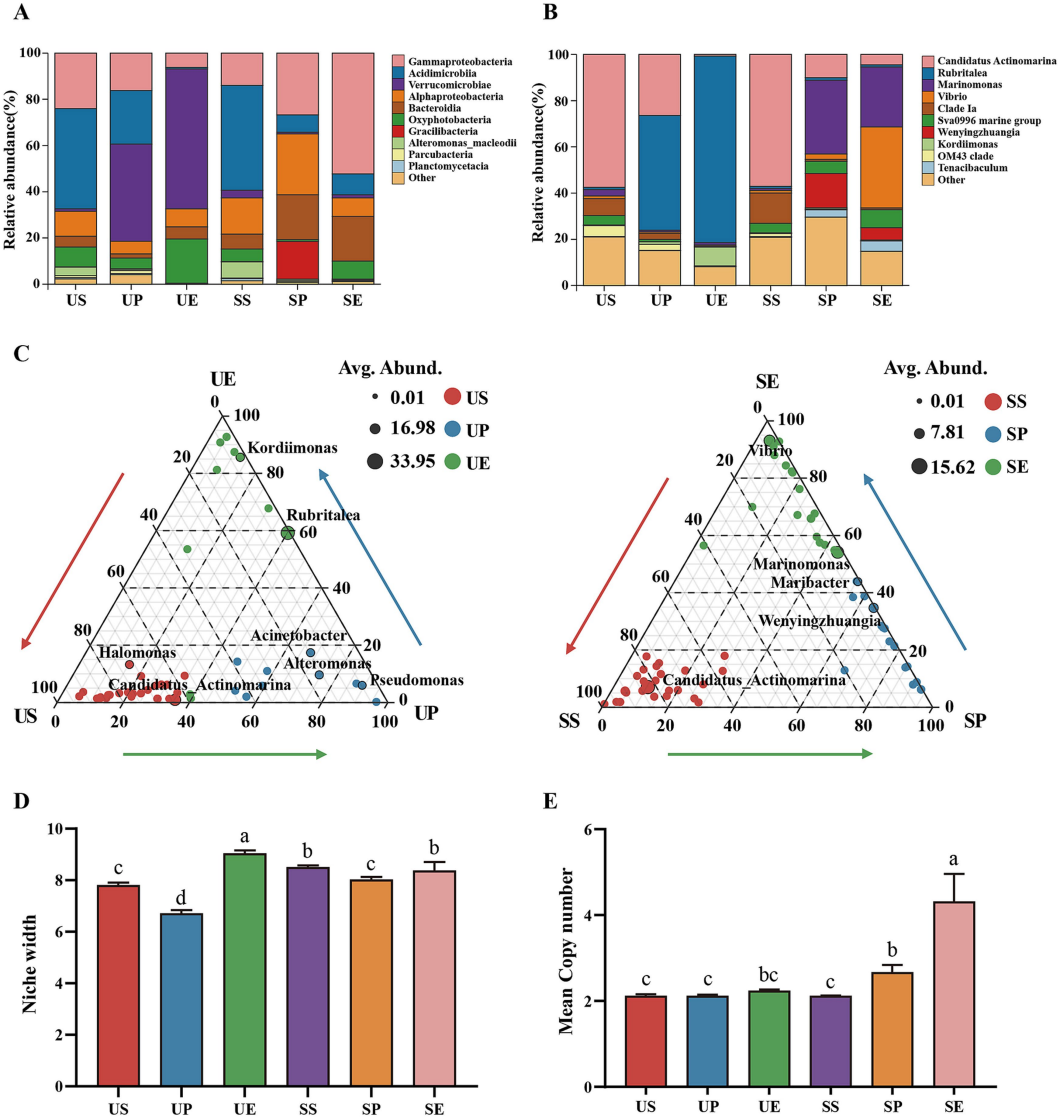
Differences in the bacterial community structure between the *U. prolifera* and *S. horneri*. Relative abundance of bacteria at the **(A)** class and **(B)** genus level. **(C)** Indicator bacteria with significant differences at the genus level (*p < 0.01*). **(D)** The niche width and **(E)** 16S rRNA genes mean copy number among the six groups. Values are represented as means ± SD (*n* = 3). Different lowercase letters indicate a significant difference among groups according to ANOVA and the LSD test (*p < 0.05*); the same lowercase letters indicate no significant difference between the groups (*p > 0.05*). US: *U. prolifera* surrounding seawater; UP: *U. prolifera* phycosphere; UE: *U. prolifera* epiphytic bacteria; SS: *S. horneri* surrounding seawater; SP: *S. horneri* phycosphere; SE: *S. horneri* epiphytic bacteria.

To characterize the differences in bacterial community structure between *U. prolifera* and *S. horneri*, we first analyzed the shared and unique OTUs. The epiphytic bacteria of both *U. prolifera* and *S. horneri* harbored the fewest unique OTUs compared to those in the phycosphere and surrounding seawater. Notably, the proportion of unique OTUs in the epiphytic bacteria of *U. prolifera* (3.63%) was substantially lower than that in *S. horneri* (13.99%) (Supplementary Figure S1B). For the bacterial composition, Verrucomicrobiae, Acidimicrobiia, and Gammaproteobacteria were dominant at the class level in the *U. prolifera* phycosphere, whereas Gammaproteobacteria, Alphaproteobacteria, and Bacteroidia were dominant in the *S. horneri* phycosphere. The dominant bacteria in the *U. prolifera* epiphytic bacteria included Verrucomicrobiae and Oxyphotobacteria, whereas those in the *S. horneri* epiphytic bacteria were Gammaproteobacteria and Bacteroidia (Figure 2A). Phycospheric and epiphytic bacterial communities were generally dominated by a few abundant genera. For example, *Rubritalea* and *Candidatus Actinomarina* were predominant in the *U. prolifera* phycosphere, and *Marinomonas* was prominent in *S. horneri* phycosphere. *Rubritalea* comprised an overwhelmingly large proportion of the *U. prolifera* epiphytic bacteria, whereas *Vibrio* and *Marinomonas* dominated the *S. horneri* epiphytic bacteria (Figure 2B).

Linear discriminant analysis effect size (LEfSe) was performed to explore the signature microbes among the groups, from the phylum level to the genus level. The LEfSe (LDA > 4) analysis identified fewer biomarkers in the phycosphere of *U. prolifera* and *S. horneri* than those in the surrounding seawater and in the epiphytic bacterial communities (Supplementary Figure S2). In addition, several biomarkers were detected with significant differences at the genus level (*p* < 0.01) (Figure 2C). The biomarkers in the surrounding seawater of both macroalgal species were strikingly similar. The dominant biomarkers in the *U. prolifera* phycosphere were *Rubritalea* and *Candidatus Actinomarina*, and those in the *U. prolifera* epiphytic bacteria were *Rubritalea* and *Kordiimonas.* For *S. horneri*, *Marinomonas* and *Wenyingzhuangia* were enriched in the phycosphere, and *Vibrio* and *Marinomonas* were prominent among the epiphytes.

The comparative analysis of niche width revealed that the epiphytic bacteria of *U. prolifera* and *S. horneri* had greater niche widths than the surrounding seawater and the phycosphere (Figure 2D). The life strategies of these bacteria were explored further by assessing the average numbers of copies of their 16S rRNA genes. The *S. horneri* epiphytic bacteria had substantially higher numbers of copies than those in other groups (Figure 2E). Together, these results demonstrate that *U. prolifera* and *S. horneri* exhibit significant differences in terms of the diversity and community structure of their phycospheric and epiphytic bacteria.

### 3.3 Influence of environmental factors and assembly processes on bacterial communities of *Ulva prolifera* and *Sargassum horneri*

**Figure 3:**
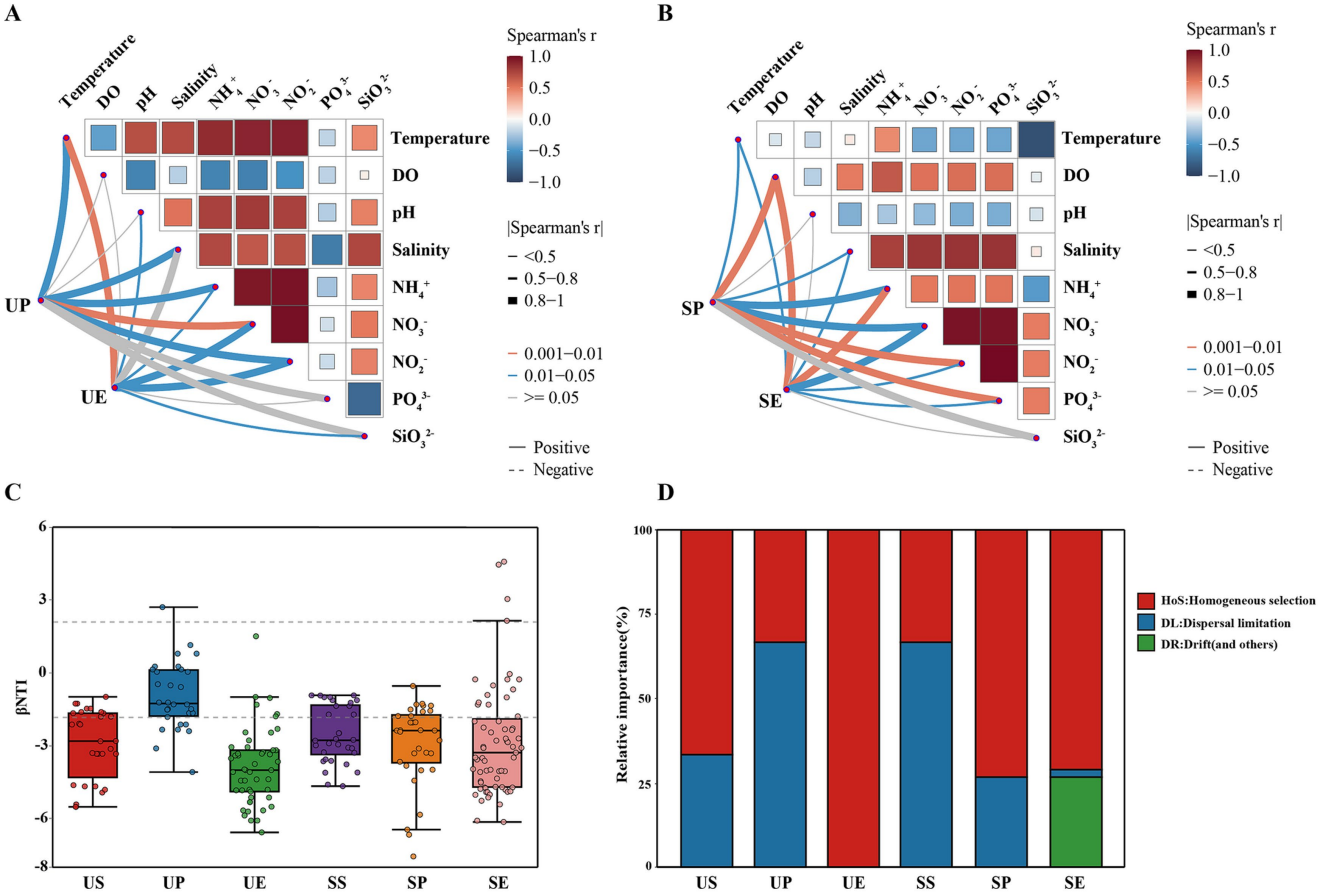
Environmental factors and ecological processes shaping bacterial community structure between the *U. prolifera* and *S. horneri*. Associations of **(A)** *U. prolifera* and **(B)** *S. horneri* bacterial community structure with environmental factors using the Mantel test. Mantel’s *r* values are indicated by the edge width, while the statistical significance is denoted by the edge color. Pairwise correlations of environmental variables are depicted with a color gradient reflecting Spearman’s correlation coefficient. **(C)** The contributions of deterministic processes (|βNTI| ≥ 2) and random processes (|βNTI| < 2) to the assembly of bacterial communities among the six groups. **(D)** Relative contributions of different ecological processes driving the assembly of bacterial communities among the six groups. US: *U. prolifera* surrounding seawater; UP: *U. prolifera* phycosphere; UE: *U. prolifera* epiphytic bacteria; SS: *S. horneri* surrounding seawater; SP: *S. horneri* phycosphere; SE: *S. horneri* epiphytic bacteria.

The Mantel test was used to examine the influence of environmental variables on bacterial community differences between *U. prolifera* and *S. horneri*. The bacterial community structure in the *U. prolifera* phycosphere was significantly influenced by NO_3_^−^, temperature, salinity, NH_4_^+^, and NO_2_^−^ (*p* < 0.05), with temperature, NO_3_^−^, and NO_2_^−^ being the primary factors for *U. prolifera* epiphytic bacteria (*p* < 0.05) (Figure 3A; Supplementary Table S1). With respect to the *S. horneri*, both the phycospheric and epiphytic bacterial communities showed significant correlations with DO, NH_4_^+^, and NO_3_^−^ (*p* < 0.05) (Figure 3B; Supplementary Table S2). The above results indicated that nutrients (particularly nitrogen compounds) were major environmental factors influencing the phycospheric and epiphytic communities of *U. prolifera* and *S. horneri.* Null models were used to further investigate the assembly processes of bacterial communities in the phycospheres of *U. prolifera* and *S. horneri*. Compared with the surrounding seawater, the assembly of epiphytic bacterial communities on *U. prolifera* and *S. horneri* was primarily governed by deterministic processes (|βNTI| ≥ 2). Moreover, homogeneous selection was the dominant process governing the assembly of the *U. prolifera* epiphytes (Figures 3C,D).

### 3.4 Metabolite divergence and association with bacterial communities between *Ulva prolifera* and *Sargassum horneri*

**Figure 4:**
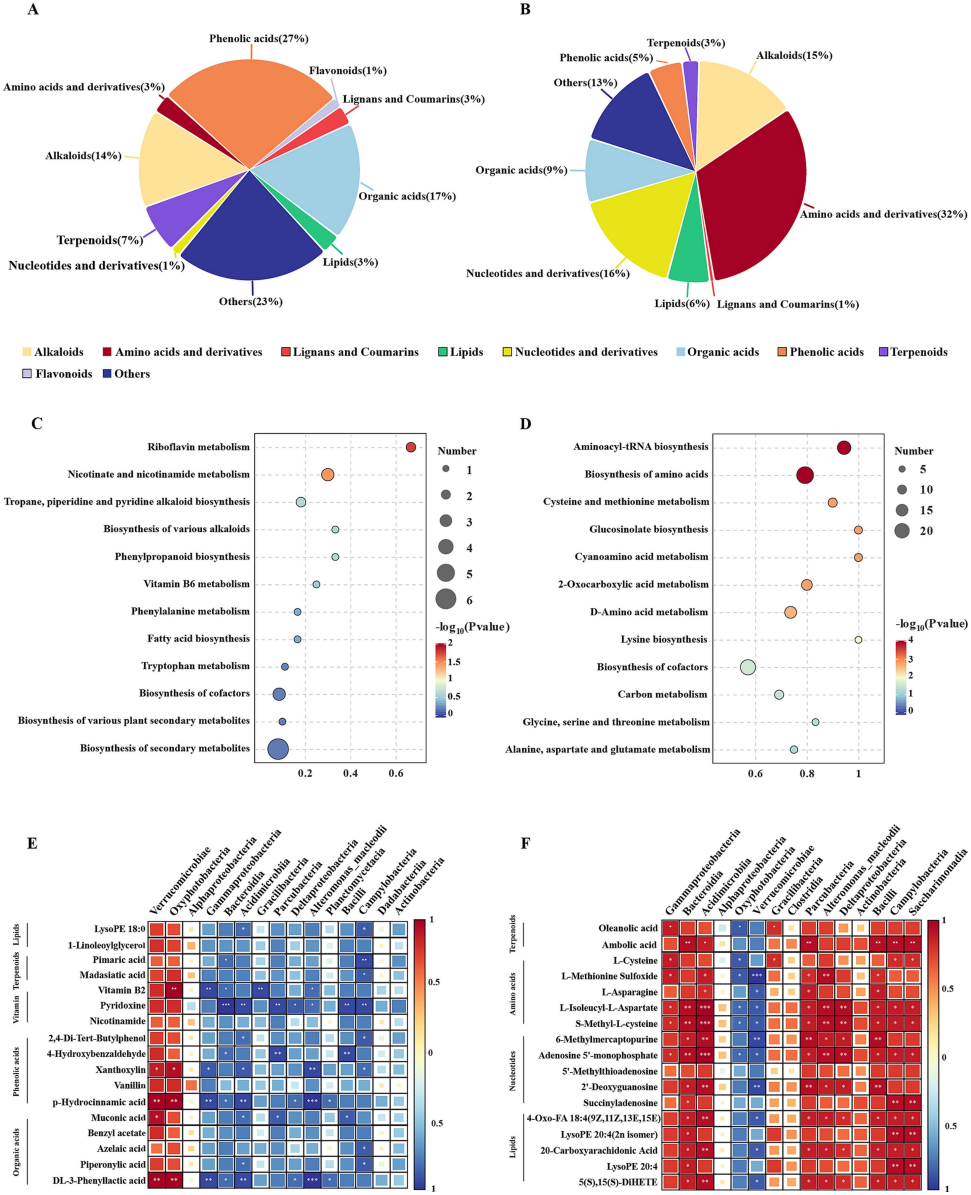
Correlation analysis between metabolites and bacteria of the *U. prolifera* and *S. horneri*. The statistical classification of the differentially metabolites in the **(A)** *U. prolifera* and **(B)** *S. horneri.* Enrichment pathways of major differentially metabolites in the **(C)** *U. prolifera* and **(D)** *S. horneri.* Correlation analysis between the top 15 most abundant bacteria at the class level and differentially metabolites in the **(E)** *U. prolifera* and **(F)** *S. horneri.* *0.01 < *p* ≤ 0.05, **0.001 < *p* ≤ 0.01, ***p ≤ 0.001.

Comprehensive metabolomic profiling of *U. prolifera* and *S. horneri* identified a total of 475 metabolites. Significant differences in metabolites were observed between *U. prolifera* and *S. horneri* based on a PCA plot (Supplementary Figure S3A). A total of 70 differentially abundant metabolites were identified in *U. prolifera*, with phenolic acids and organic acids being the most abundant metabolites, along with detectable levels of flavonoids (Figure 4A). In contrast, 160 differentially abundant metabolites were detected in *S. horneri*, predominantly comprising amino acids and derivatives, nucleotides and derivatives, and alkaloids (Figure 4B). In *U. prolifera*, the major differential metabolites were significantly enriched in the “riboflavin metabolism” and “nicotinate and nicotinamide metabolism” pathways (Figure 4C). For *S. horneri*, “aminoacyl-tRNA biosynthesis” and “biosynthesis of amino acids” were the most significantly enriched pathways (Figure 4D).

The associations between the algal metabolome and the bacterial communities were also investigated. Procrustes analysis indicated a significant overall association between the phylum-level bacterial microbiome and the metabolome in *U. prolifera* and *S. horneri* (*M*^2^ = 0.081, *p* = 0.035) (Supplementary Figure S3B). A heatmap was generated based on Spearman’s correlation tests to elucidate the specific relationships between metabolites and bacteria. Verrucomicrobiae and Oxyphotobacteria were positively associated with various metabolites in *U. prolifera*, such as phenolic acids, organic acids, vitamins, and terpenoids (Figure 4E). In *S. horneri*, Bacteroidia, Acidimicrobiia, Parcubacteria, Deltaproteobacteria, Bacilli, Campylobacteria, and Saccharimonadia showed significant and positive associations with various metabolites, such as amino acids, nucleotides, and terpenoids (*p* < 0.05) (Figure 4F).

## 4 Discussion

Algal bloom dynamics are regulated by a complex interplay between environmental factors and the algae-associated microbiome (Zhou et al., 2020a). However, increasing studies have demonstrated that algae–microbial interactions predominantly determine bloom formation and development (Cui et al., 2020), thus highlighting the critical role of microbial community structures in bloom progression. Phycospheric and epiphytic bacterial communities provide a relatively “focused” perspective for understanding host-microbe relationships and characterizing the functional importance of microbial communities (Lu et al., 2023; Yue et al., 2025). However, there is still limited understanding regarding whether each macroalga hosts characteristic microbiomes and the relationships regulating macroalgae–bacteria interactions. Several studies have shown that a complex host morphological structure can enhance microbial diversity (Guo et al., 2022). *Sargassum* exhibits relatively complex morphological characteristics, such as distinct lateral branches, leaves, and reproductive receptacles, whereas *Ulva* sp. are characterized by relatively simple tubular or filiform structures (Hervé et al., 2021). Such morphological complexity may partially explain why *S. horneri* blooms exhibit higher Shannon diversity indices in their phycospheric and epiphytic bacterial communities than *U. prolifera* blooms (Figures 1B,C). In addition, the growth duration of macroalgae may also be critical for shaping microbial diversity. Previous studies have indicated that macroalgae with longer growth periods tend to accumulate more mature and highly colonized microbial communities than those with shorter lifespans (Lemay et al., 2018; Weigel and Pfister, 2019). Given that *S. horneri* has a substantially longer growth period, while *U. prolifera* is known for its rapid growth and short life cycle, this further explains why *S. horneri* exhibited a significantly higher Shannon diversity. This is further supported by the OTU profiles, which revealed that *U. prolifera* had the fewest unique OTUs (Supplementary Figure S1B).

Macroalgal surfaces often have unique microbial communities that differ from those in the surrounding seawater (Quigley et al., 2020), although some bacterial groups are common among various macroalgae hosts (Florez et al., 2017). This is attributed to the specific microenvironmental conditions and ecological niches provided by macroalgae, which are conducive to the proliferation of bacterial taxa with distinct functional capabilities (Nahor et al., 2024). Similarly, the present study revealed significant differences in *β* diversity in the epiphytic and phycospheric bacterial communities on *U. prolifera* and *S. horneri* compared with the surrounding seawater (Supplementary Figure S1A). The excessive proliferation or aggregation of *U. prolifera* and *S. horneri* released nutrients, thereby creating favorable conditions for bacterial colonization on the thallus surfaces. Differences in the released nutrients may have partially influenced the dispersion of bacteria, resulting in the selective enrichment of microbial communities adapted to specific habitats (Yang et al., 2025). Notably, Verrucomicrobiae enrichment was observed in phycospheric and epiphytic bacterial colonies on *U. prolifera* (Figure 2A), which can degrade diverse polysaccharides (Martinez-Garcia et al., 2012) and fix nitrogen (Wertz et al., 2012). Although Verrucomicrobia exhibit metabolic diversity, they are frequently associated with carbohydrate degradation in aquatic ecosystems (Tran et al., 2018), particularly cellulose and chitin polymers, reflecting adaptations to the availability of carbon substrates on *U. prolifera* (Parveen et al., 2013). Similarly, significant Bacteroidia enrichment on *S. horneri*, which can be associated with the utilization of polysaccharides, such as fucose, laminarin, and rhamnose (Orellana et al., 2022), explains why they are commonly found in the microbial assemblages of *S. horneri* blooms. Analysis of the indicator species further highlighted these contrasts (Figure 2C). Verrucomicrobial *Rubritalea* are known to feature biofilm-forming bacteria and were the most abundant biomarkers in the phycospheric and epiphytic bacterial communities of *U. prolifera*. This finding is consistent with previous reports identifying *Rubritalea* as a stable core component in the phycosphere of *Ulva* sp. (Lu et al., 2023). Meanwhile, Gammaproteobacteria *Marinomonas* were identified as a key biomarker for *S. horneri*, consistent with its abundance in the epiphytes of intertidal brown macroalgae (Chen et al., 2022). These species-specific biomarkers provide potential ecological biomarkers for tracking and assessing the dynamics of their respective bloom events.

During both *U. prolifera* and *S. horneri* blooms, the niche breadth of epiphytic bacteria was higher than that of the phycosphere and surrounding seawater (Figure 2D). This phenomenon can likely be attributed to the capacity of macroalgal secretions to directionally and selectively enrich microbial taxa that fulfill specific algal requirements (Brisson et al., 2023). Such selective pressures facilitate broad resource utilization and enhance environmental adaptability among epiphytes, thereby expanding their niche breadth. This hypothesis is supported by substantial evidence that epiphytes associated with hosts, such as macroalgae and seagrasses, generally exhibit higher metabolic diversity than seawater microorganisms (Chen et al., 2022; Lu et al., 2023). Moreover, the significantly higher 16S rRNA operon copy number in the epiphytic bacteria of *S. horneri* compared to that of *U. prolifera* is consistent with an r-selected life-history strategy (Figure 2E), which favors rapid growth under resource-rich conditions (Wu et al., 2017). Therefore, *S. horneri* and *U. prolifera* have microecosystems that appear to increase their survival requirements by recruiting epiphytes with diverse life-history strategies (Roller et al., 2016). This likely provides an ecological foundation for the development and persistence of macroalgal blooms. Additionally, studies have reported that changes in bacterial life-history strategies may be influenced by the algal reproductive period or direct responses to seasonal environmental changes (Hall and MacPherson, 2025). Therefore, further studies are needed to validate these findings.

Differences in the phycosphere community compositions of *U. prolifera* and *S. horneri* reflect the distinct characteristics of their colony microenvironments. Therefore, we examined the effects of environmental variables on bacterial communities during the bi-macroalgal blooms. In this study, nutrients, especially NH_4_^+^, NO_3_^−^, and NO_2_^−^, emerged as the primary predictors of bacterial assemblies in both phycospheric and epiphytic bacterial communities (Figures 3A,B). Nutrients are key factors influencing the bacterial community structure (Li S. et al., 2025); specifically, they exert environmental selection pressures on bacterial structures by shaping bacterial growth constraints and modifying resource availability within microenvironments (Kalra et al., 2025). Furthermore, the phycospheric and epiphytic bacterial community structures of *S. horneri* were correlated with DO (Figure 3B), which is associated with the decline stage of *S. horneri* blooms. The decomposition of *S. horneri* would usually have depleted the DO from seawater, thereby creating niches suitable for facultative or obligate anaerobes (Broman et al., 2017; Li et al., 2024). Despite the effects of environmental factors (e.g., selection) on bacterial community structures, the null model analysis provided compelling evidence that deterministic processes predominantly govern the assembly mechanisms of bacterial communities during *U. prolifera* and *S. horneri* blooms. Previous studies showed that microorganisms in open waters are more strongly affected by random factors, such as water currents and biological migration, which limit sustained selection pressures (Zhou et al., 2014; Langenheder and Lindström, 2019). However, the present study demonstrated that deterministic processes prevail in the assembly of bacterial communities during bi-macroalgal blooms (Figure 3C). Notably, the proportion of deterministic processes was significantly higher in the epiphytes (Figure 3D). This phenomenon was likely due to the strong environmental filtering of bacterial communities by macroalgae, which modified microhabitat conditions to select for adapted bacterial taxa (Zhou et al., 2020b). This active filtering effect enhanced the deterministic contribution to the structuring of epiphytic bacterial communities. However, simple amplicon sequencing can only indirectly infer these assembly mechanisms; therefore, future studies should employ multi-omics approaches to produce more plausible imputations and more reliable inferences.

Bacterial community variations are due not only to changes in the physicochemical environment but also to host-derived metabolites that collectively regulate bacterial variability. Studies of epiphytic bacteria from different macroalgae have consistently shown that their diversity is primarily influenced by algal secondary metabolites (Sneed and Pohnert, 2011; Quigley et al., 2020), suggesting that macroalgae can exert selective pressures on bacterial communities through the secretion of specific metabolites (Roth-Schulze et al., 2016; Selvarajan et al., 2019). This process represents an active recruitment strategy in which macroalgae attract beneficial microbes via thallus exudates. Metabolome analysis showed that the contents of phenolic acids, flavonoids, and organic acids were significantly greater in *U. prolifera* than in *S. horneri* (Figures 4A,B). Meanwhile, Verrucomicrobiae and Oxyphotobacteria, which are the core bacterial taxa of *U. prolifera*, showed significantly positive correlations with these metabolites (Figure 4E), indicating that host-secreted metabolites can attract associated bacteria to colonize. Verrucomicrobiae *Rubritalea* can degrade algal polysaccharides with sulfatases and use the resulting carbon sources (e.g., glucose) for efficient carotenoid synthesis (Shindo et al., 2007). These carotenoids contribute to photoprotection and antioxidant defense while increasing algal stress tolerance. In contrast, the exudates of *S. horneri* contained significantly higher concentrations of amino acids and nucleotides than those of *U. prolifera* (Figures 4A,B). Accordingly, the epiphytic bacterial communities of *S. horneri* were dominated by Gammaproteobacteria, Bacteroidia, Acidimicrobiia, Deltaproteobacteria, Parcubacteria, and Saccharimonadia, which showed strong correlations with the metabolism of those metabolites (Figure 4F). Bacteroidia and Deltaproteobacteria can produce alginases and fucosidases (Kim et al., 2016; Sichert et al., 2020) that allow them to derive carbon from the digestion of brown algal cell walls (Ivanova et al., 2002). Owing to a lack of complete biosynthetic pathways, some microorganisms must rely on uptaking metabolites produced by other microorganisms in the environment to maintain growth. For example, reduced-genome taxa, such as Parcubacteria (Nelson and Stegen, 2015) and Saccharimonadia (Lemos et al., 2019), exhibited significant and positive correlations with a range of fundamental nutrients on the surfaces of *S. horneri* (Figure 4F). Further analysis revealed that the most abundant Gammaproteobacteria in this study were dominated by two genera: *Vibrio* and *Marinomonas* (Figure 2B). These taxa are widely recognized as algicidal bacteria capable of lysing algal cells through the secretion of algicidal enzymes and extracellular compounds (Yu et al., 2020; Yu et al., 2024). Such algicidal activity contributes to the dynamics of *S. horneri* blooms and the marine carbon cycle. Host-derived metabolites shape microbiome composition through direct interactions while also serving as signaling molecules that regulate microbial dynamics (Ilyaskina et al., 2025). Previous studies have identified specific components involved in chemical communication, including flavonoids and terpenoids (Jacoby et al., 2017). In this study, terpenoids secreted by both *U. prolifera* and *S. horneri* were positively correlated with most bacterial taxa (Figures 4E,F). These terpenoids may function as signaling molecules in quorum sensing or as chemoattractants that facilitate the selective colonization of bacteria on thalli (Singh and Reddy, 2014). Consequently, it is reasonable to propose that the specific molecules found in macroalgal exudates help shape the structure of the microbial community.

## 5 Conclusion

The findings presented in this study are the first to examine the characteristics of phycosphere-associated bacterial communities and algae-derived metabolites between the *U. prolifera* and *S. horneri* blooms. The diversity and composition of phycospheric and epiphytic bacterial communities were significantly different between *U. prolifera* and *S. horneri*. Verrucomicrobiae were the core components of the *U. prolifera* phycosphere, whereas Gammaproteobacteria and Bacteroidia were the core components of the *S. horneri* phycosphere. Deterministic processes influenced the assembly of epiphytic bacterial communities during the bi-macroalgal blooms. Composition differences in metabolite secretion underpin host-specific microbial recruitment between *U. prolifera* and *S. horneri*. Secreted metabolites of *U. prolifera* (e.g., phenolic acids and organic acids) promoted the colonization of Verrucomicrobiae and Oxyphotobacteria, thereby enhancing host stress tolerance. In contrast, the key metabolites in *S. horneri* exudates (e.g., amino acids, nucleotides, and lipids) facilitated the colonization of algicidal *Vibrio* and *Marinomonas*, potentially inhibiting host growth. Overall, the results demonstrate that macroalgae species secrete distinct metabolites to recruit specific microbial communities, thereby differentially influencing host-bacteria interactions.

## Data Availability

The raw data of this article are available in the National Center for Biotechnology Information (NCBI) repository, and is available under PRJNA1348149.

## Footnotes

1
www.r-project.org
