# Supplementary material for: A comparative analysis of bacterial community characterization and host–bacteria interactions between bi-macroalgal blooms caused by Ulva prolifera and Sargassum horneri
Source: Front Microbiol. 2025 Dec 18;16:1728378. doi: 10.3389/fmicb.2025.1728378 (PMC12756825; doi:10.3389/fmicb.2025.1728378)
Supplement: Supplementary file 1 [file Data_Sheet_1.docx]

Supplementary Material

# Supplementary Figures and Tables

## Supplementary Figures


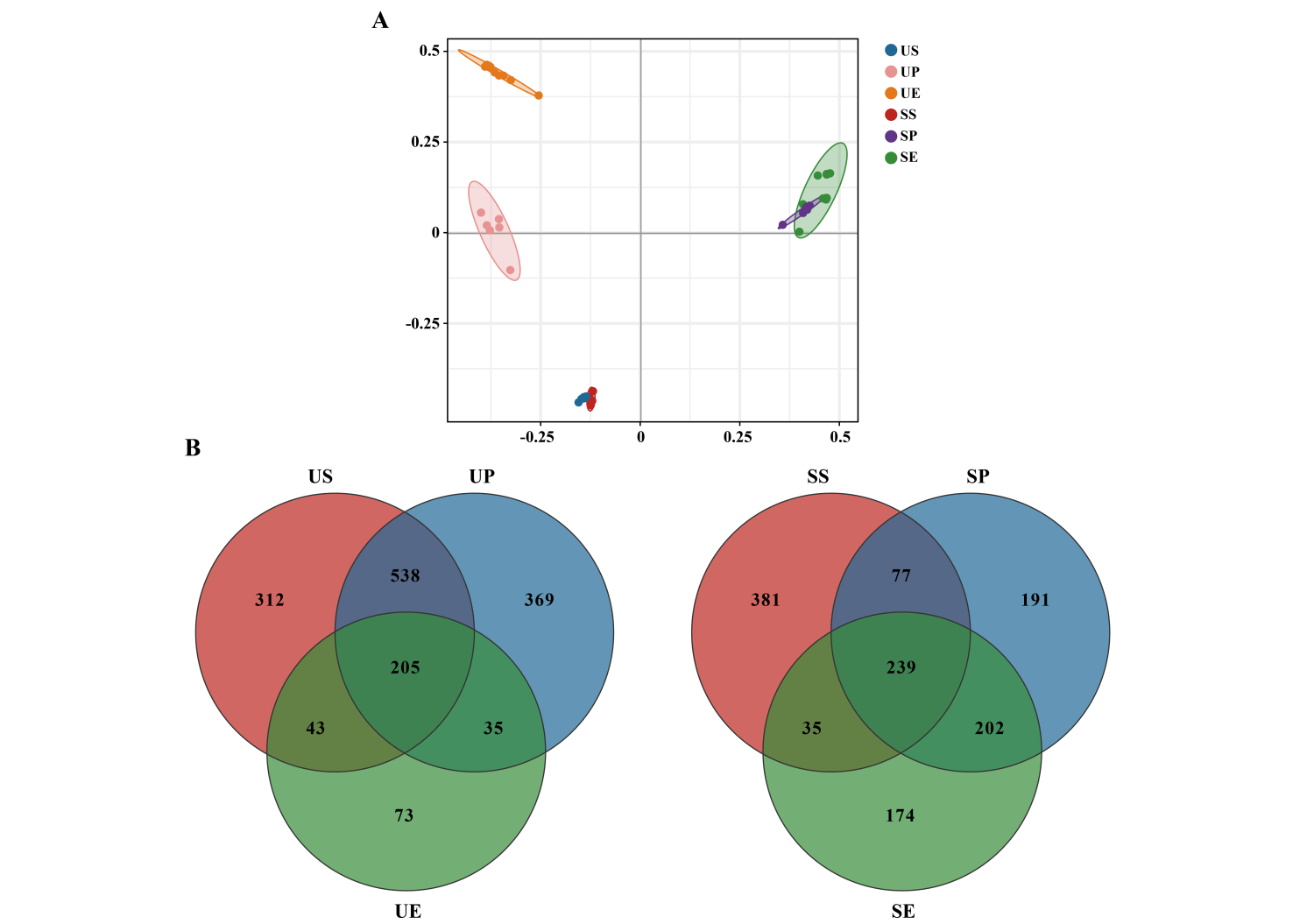


**Supplementary Figure 1. (A)** PCoA of bacterial communities at the OTU level between the *U. prolifera* and *S. horneri*. **(B)** Venn diagram illustrating the shared and unique bacterial OTUs among the six groups. 95% confidence ellipses are shown around the samples and grouped based on the six groups. US: *U. prolifera* surrounding seawater; UP: *U. prolifera* phycospheric seawater; UE: *U. prolifera* epiphytic bacteria; SS: *S. horneri* surrounding seawater; SP: *S. horneri* phycospheric seawater; SE: *S. horneri* epiphytic bacteria.


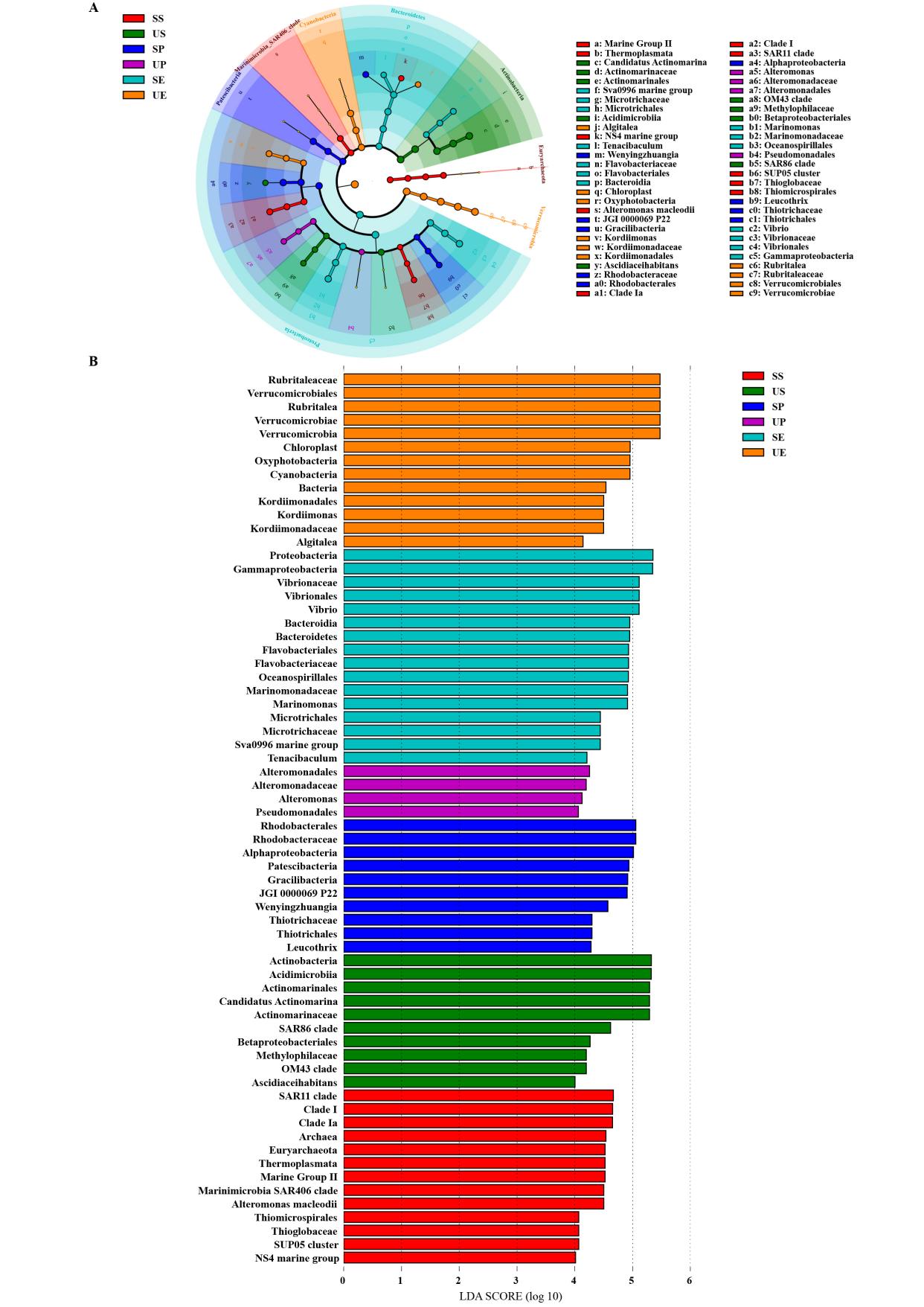


**Supplementary Figure 2.** Biomarkers analysis of bacteria between the *U. prolifera* and *S. horneri*. **(A)** Cladogram shows the phylogenetic structures of bacteria. **(B)** LDA score chart indicate significant differences in bacterial taxa (LDA score >4.0). US: *U. prolifera* surrounding seawater; UP: *U. prolifera* phycospheric seawater; UE: *U. prolifera* epiphytic bacteria; SS: *S. horneri* surrounding seawater; SP: *S. horneri* phycospheric seawater; SE: *S. horneri* epiphytic bacteria.

# Supplementary Table 1. Mantel test of *U. prolifera* bacterial community structure with environmental factors: Correlation Coefficient (r) and p-value (P).

| **Group** | **Environmental factors** | **r** | **P** |
| --- | --- | --- | --- |
| *U. prolifera* phycospheric seawater | Temperature | 0.964 | 0.043 |
| *U. prolifera* phycospheric seawater | pH | 0.082 | 0.225 |
| *U. prolifera* phycospheric seawater | DO | 0.385 | 0.138 |
| *U. prolifera* phycospheric seawater | Salinity | 0.878 | 0.047 |
| *U. prolifera* phycospheric seawater | NH_4_^+^ | 0.851 | 0.011 |
| *U. prolifera* phycospheric seawater | NO_3_^-^ | 0.987 | 0.008 |
| *U. prolifera* phycospheric seawater | NO_2_^-^ | 0.873 | 0.035 |
| *U. prolifera* phycospheric seawater | PO_4_^3-^ | 0.871 | 0.092 |
| *U. prolifera* phycospheric seawater | SiO_3_^2-^ | 0.970 | 0.058 |
| *U. prolifera* epiphytic bacteria | Temperature | 0.964 | 0.008 |
| *U. prolifera* epiphytic bacteria | DO | 0.184 | 0.050 |
| *U. prolifera* epiphytic bacteria | pH | 0.758 | 0.021 |
| *U. prolifera* epiphytic bacteria | Salinity | 0.817 | 0.092 |
| *U. prolifera* epiphytic bacteria | NH_4_^+^ | 0.720 | 0.013 |
| *U. prolifera* epiphytic bacteria | NO_3_^-^ | 0.979 | 0.022 |
| *U. prolifera* epiphytic bacteria | NO_2_^-^ | 0.973 | 0.022 |
| *U. prolifera* epiphytic bacteria | PO_4_^3-^ | 0.021 | 0.367 |
| *U. prolifera* epiphytic bacteria | SiO_3_^2-^ | 0.507 | 0.014 |

# Supplementary Table 2. Mantel test of *S. horneri* bacterial community structure with environmental factors: Correlation Coefficient (r) and p-value (P).

| **Group** | **Environmental factors** | **r** | **P** |
| --- | --- | --- | --- |
| *S. horneri* phycospheric seawater | Temperature | 0.607 | 0.031 |
| *S. horneri* phycospheric seawater | DO | 0.858 | 0.001 |
| *S. horneri* phycospheric seawater | pH | 0.106 | 0.374 |
| *S. horneri* phycospheric seawater | Salinity | 0.758 | 0.043 |
| *S. horneri* phycospheric seawater | NH_4_^+^ | 0.993 | 0.043 |
| *S. horneri* phycospheric seawater | NO_3_^-^ | 0.993 | 0.017 |
| *S. horneri* phycospheric seawater | NO_2_^-^ | 0.995 | 0.003 |
| *S. horneri* phycospheric seawater | PO_4_^3-^ | 0.990 | 0.003 |
| *S. horneri* phycospheric seawater | SiO_3_^2-^ | 0.982 | 0.078 |
| *S. horneri* epiphytic bacteria | Temperature | 0.581 | 0.036 |
| *S. horneri* epiphytic bacteria | DO | 0.896 | 0.001 |
| *S. horneri* epiphytic bacteria | pH | 0.041 | 0.353 |
| *S. horneri* epiphytic bacteria | Salinity | 0.758 | 0.018 |
| *S. horneri* epiphytic bacteria | NH_4_^+^ | 0.985 | 0.001 |
| *S. horneri* epiphytic bacteria | NO_3_^-^ | 0.925 | 0.035 |
| *S. horneri* epiphytic bacteria | NO_2_^-^ | 0.574 | 0.035 |
| *S. horneri* epiphytic bacteria | PO_4_^3-^ | 0.599 | 0.036 |
| *S. horneri* epiphytic bacteria | SiO_3_^2-^ | 0.296 | 0.126 |

**
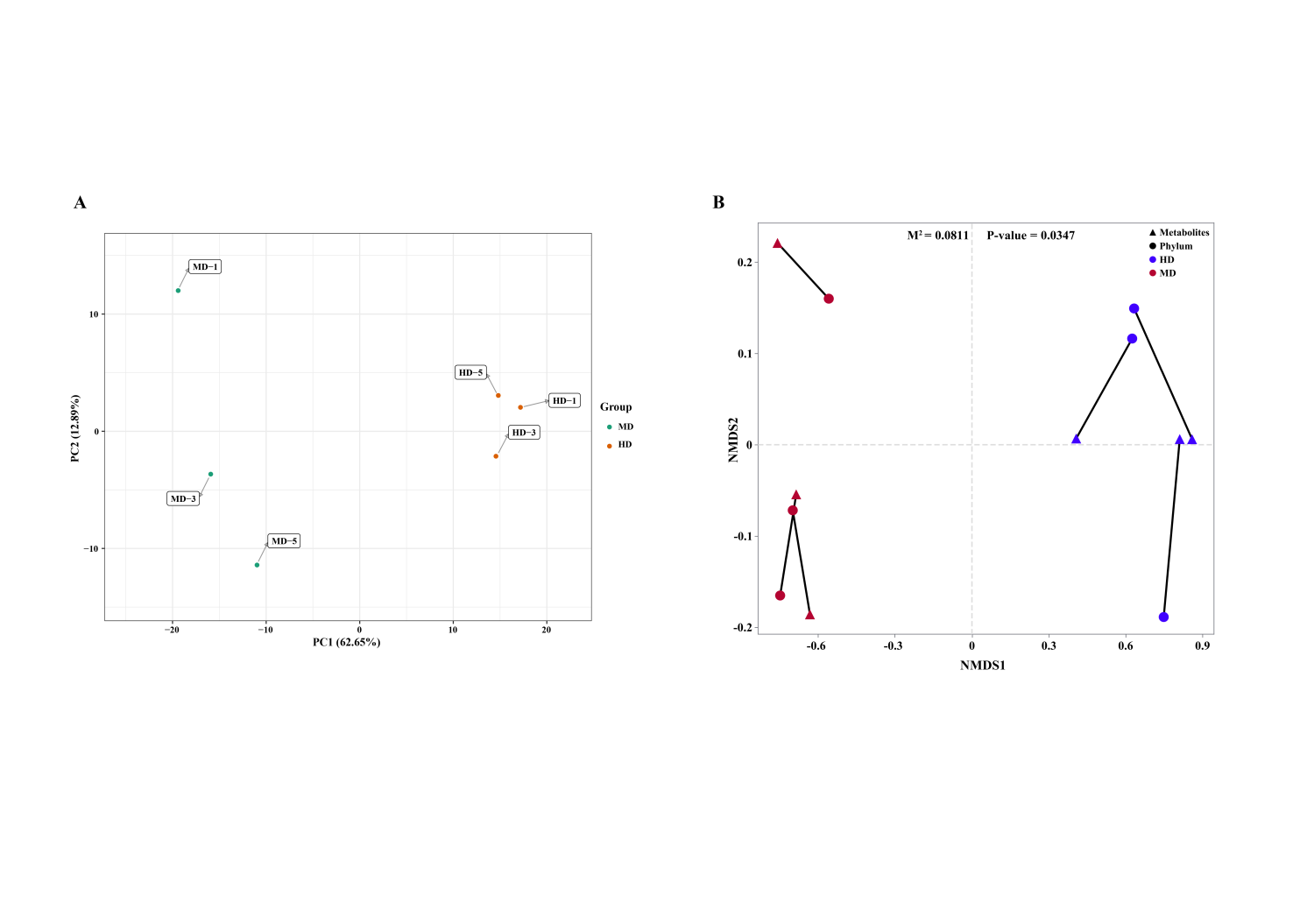
**

**Supplementary Figure 3. (A)** PCA of metabolites of *U. prolifera* and *S. horneri.* **(B)** Procrustes analysis of the correlation between bacterial community at the phylum level and metabolites profiles in the *U. prolifera* and *S. horneri*. HD: *U. prolifera*; MD: *S. horneri*.
